# Refus et hésitation vis-à-vis de la vaccination anti-COVID-19 à Douala, Cameroun

**DOI:** 10.11604/pamj.2024.48.61.39880

**Published:** 2024-06-14

**Authors:** André Arsène Bita Fouda, Verdiane Félécité Metagne Kengne, Dieudonné Adiogo, Léon Jules Owona Manga

**Affiliations:** 1 Faculté de Médecine et de Sciences Pharmaceutiques de Douala, Douala, Cameroun

**Keywords:** Hésitation vaccinale, refus vaccinal, vaccination, COVID-19, Douala, Cameroun, Vaccine refusal, vaccine hesitation, vaccination, COVID-19, Douala, Cameroon

## Abstract

**Introduction:**

la vaccination est l'une des stratégies recommandées par l'Organisation mondiale de la Santé pour réduire le poids de la COVID-19. Cependant, de nombreux pays africains comme le Cameroun présentent de faibles couvertures vaccinales anti-COVID-19. Cette étude avait pour objectif d'étudier les raisons de refus de la population de la ville de Douala vis-à-vis de la vaccination contre la COVID-19.

**Méthodes:**

l'étude était transversale et analytique et conduite dans la ville de Douala du 10 février au 31 mai 2022. Les participants étaient âgés d'au moins 21 ans résidant dans la ville de Douala étaient interviewés. Les mesures d'association entre les variables d'intérêt ont été effectuées à l'aide des tests de Chi-deux et de Fisher avec un intervalle de confiance à 95%.

**Résultats:**

au total, 1555 personnes avaient été incluses dans l'étude. Seulement 168 (11%) étaient vaccinées. La proportion de refus vaccinal était importante 711 (45,7%), 640 participants soit 41,1% hésitaient alors que 204 personnes soit 13,2% étaient favorable à la vaccination anti-COVID-19. Les raisons principales de refus de vaccination anti-COVID-19 étaient la crainte des effets indésirables 406 (44,8%), le manque d'information sur les vaccins 331 (36,5%) et le manque de confiance 302 (33,3%). Les facteurs associés au refus vaccinal étaient la religion (p=0,026) et le niveau d'étude (p=0,002).

**Conclusion:**

cette étude avait révélé la faible couverture vaccinale anti-COVID-19 à Douala avec une proportion importante de refus et hésitation vaccinale. Les stratégies de communication devraient tenir compte des raisons et facteurs associés au refus.

## Abstract (en)

**Introduction:**

vaccination is one of the strategies the World Health Organization recommends to reduce the burden of COVID-19. However, many African countries like Cameroon have low COVID-19 vaccination coverage. The purpose of this study was to investigate the reasons for the refusal of the population of the city of Douala to be vaccinated against COVID-19.

**Methods:**

we conducted a cross-sectional and analytical study in Douala from February 10 to May 31, 2022. Participants, aged at least 21 years and residing in the city of Douala, were interviewed. Associations between the variables of interest were measured using Chi-square and Fisher tests, with a 95% confidence interval.

**Results:**

a total of 1555 people were included in the study. Only 168 (11%) had been vaccinated. The proportion of vaccine refusal was high, with 711 (45.7%) refusing, 640 participants (41.1%) hesitating, and 204 people (13.2%) being in favor of COVID-19 vaccination. The main reasons for refusing anti-COVID-19 vaccination were fear of adverse effects (406; 44.8%), lack of information about vaccines (331; 36.5%), and lack of confidence (302; 33.3%). Factors associated with vaccine refusal were religion (p=0.026) and level of education (p=0.002).

**Conclusion:**

this study revealed low COVID-19 vaccination coverage in Douala, with a significant proportion of refusal and hesitation towards vaccination. Communication strategies should take into account the reasons and factors associated with refusal.

## Introduction

Depuis l'apparition de la COVID-19 à Wuhan en Chine au cours du dernier trimestre de l'année 2019, plus de 6 millions de morts ont été enregistrés dans le monde [1,2]. La COVID-19 est ainsi devenue le principal problème de santé publique dans le monde. Elle présente un risque élevé pour les pays ayant des systèmes de santé vulnérables [2]. Son taux de mortalité variant entre 0,5 et 3%, augmente avec l'âge et la présence de comorbidités telles que l'hypertension artérielle, le diabète, les coronaropathies, les pathologies respiratoires chroniques, l'insuffisance rénale chronique, les cancers et l'obésité sévère [3].

À la date du 31 juin 2022, l'Organisation mondiale de la Santé (OMS) déclarait 545.226.550 cas de COVID-19 et 6.334.728 décès, soit un taux de mortalité de 1,2% dans le monde [1]. La morbidité et la mortalité sont plus marquées dans les pays développés. Quant au continent africain, 11.965.697 de cas et 253.119 décès y ont été enregistrés à la date du 31 juin 2022, soit un taux de mortalité de 2,2%. Les pays les plus affectés étaient l'Afrique du Sud avec 3.997.979 cas et 101.002 décès, le Maroc avec 1.189.660 et 16.075 décès et la Tunisie 1.046.709 cas et 28.632 décès. Le Cameroun comptait à la même date 119.947 cas confirmés et 1.930 décès pour un taux de mortalité de 1,6% [4]. Toutefois, il serait probable que le nombre réel d'infections soit plus élevé, car le nombre de tests réalisés dans plusieurs pays est faible et plusieurs personnes asymptomatiques n'ont probablement pas été diagnostiquées [5].

Pour stopper la progression du virus ainsi que ses conséquences sur la santé et l'économie mondiale, de nombreuses mesures préventives ont été prescrites par l'OMS et ont été mises en place dans chaque pays. Parmi ces mesures figure la vaccination dont il existe plusieurs types et plusieurs fabricants [6]. En effet, l'émergence de cette pandémie a généré un intérêt sans précédent pour le développement de vaccins permettant de protéger la population et ultimement, de contrôler cette pandémie associée à un fardeau sanitaire et socio-économique sans précédent depuis la grippe espagnole survenue au début du vingtième siècle [7,8]. Depuis son introduction, la vaccination anti-COVID-19 fait face à de nombreuses critiques pouvant être liées à sa constitution, à ses effets secondaires ou à sa sécurité. En 2019, l'OMS déclarait l'hésitation vaccinale comme une des dix menaces à la santé globale [9]. Certains auteurs ont montré que l'hésitation contre le vaccin anti-COVID-19 est plus importante comparé aux autres vaccins existants [10].

Les doutes et craintes liés à la vaccination anti-COVID-19 ne cessent de prospérer dans notre contexte, entrainant moins d'intention de se faire vacciner contre la COVID-19, plus de refus vaccinal ou d'hésitation vaccinale observés dans certains pays dans le monde dont le Cameroun [10-26]. L'OMS rapportait qu'à la date du 31 juin 2022, une faible proportion des Africains était vaccinée seulement 539.035.014 doses de vaccins anti-COVID-19 ont été administrées dans les pays africains sur un total de 11.986.040.938 doses de vaccins administrés dans le monde [27]. Au Cameroun, le rapport du Ministère de la Santé publique du 31 juin 2022, révélait que le taux de vaccination national était de 11%, soit 1.175.911 personnes, dont 140.024 dans la région du Littoral (6,5%) [28]. Les personnes ayant une comorbidité avec la COVID-19 font partir des groupes à risques devant bénéficier de la vaccination contre la COVID-19. Les statistiques de morbidité sont variables dans le monde, plus marquées dans les pays développés par rapports aux pays pauvres [29].

Au début janvier 2021, selon la liste de l'OMS, 237 candidats-vaccins contre le SARS-CoV-2 étaient en cours de développement, dont 64 en phase de développement clinique, faisant appel à huit plateformes technologiques différentes constituées des vaccins vivants atténués et des vaccins inactivés, des vaccins à base de sous-unités protéiques, des vaccins à partir du matériel génétique viral (ARN et ADN), des vaccins par vecteur viral réplicatif ou non réplicatif, et des vaccins à pseudo-particules virales. A la date du 31 juin 2022, l'OMS avait enregistré plus de 11.811.627.599 doses de vaccins administrées dont 539.035.014 en Afrique [30]. Au Cameroun, la vaccination anti-COVID-19 a été lancée au Cameroun le 12 avril 2021 et à la date du 31 juin 2022, 1.180.721.178 personnes complètement vaccinées soit 8,5% de la population cible [28].

La situation sur le refus et l'hésitation des populations camerounaises n'est pas connue. Ce travail vise à contribuer à une meilleure compréhension des refus et hésitations des populations de la ville de Douala vis-à-vis de la vaccination contre la COVID-19. Les objectifs de cette étude étaient de déterminer la couverture vaccinale des participants, de déterminer l'ampleur du refus vaccinal, de l'hésitation vaccinale et de l'intention vaccinale. Également d'identifier les raisons de refus vaccinal et les facteurs associés au refus vaccinal. La faible couverture vaccinale contre la COVID-19 au Cameroun pourrait s'expliquer par une forte ampleur du refus, de l'hésitation et de l'intention de la population de Douala vis-à-vis de la vaccination contre la COVID-19.

## Méthodes

**Cadre de l'étude:** l'étude s'était déroulée pendant 4 mois, du 10 février au 31 mai 2022 dans les aires de santé de cinq districts de santé de la ville de Douala. Douala est la capitale économique du Cameroun, peuplée d'environ 3.390.542 habitants.

**Type d'étude:** l'étude était transversale et analytique.

**Participants de l'étude:** la taille minimum calculée avec la formule de Lorentz était de 1500. Nous avons procédé à un échantillonnage probabiliste par grappes à 3 niveaux. Au premier niveau, nous avons sélectionné aléatoirement cinq sur les dix districts de santé à savoir Bangué, Cité des palmiers, Deido, Logbaba et New-Bell. Au deuxième niveau, nous avons tiré au moins 25% des aires de santé dans chaque district de santé choisi. Pour chaque aire de santé choisie, nous avons calculé proportionnellement la taille minimale, en fonction de la taille minimale de l'échantillon. Au troisième niveau, nous avons sélectionné aléatoirement les participants dans les aires de santé. Finalement 3532 personnes résidentes de la ville Douala âgé de 21 ans ont été sélectionnées.

**Collecte et analyse des données:** la collecte des données s'était déroulée du 13 mars au 25 mai . Les données étaient collectées dans un questionnaire prétesté à travers les interviews individuels conduits par des investigateurs formés et supervisés afin d'éviter des biais d'information et de sélection.

Pour les analyses statistiques nous avons utilisé les variables quantitatives et qualitatives indépendantes dont les caractéristiques sociodémographiques (âge, sexes, niveau scolaire, profession, lieu de résidence, statut matrimonial région d'origine, religion) et caractéristique clinique (comorbidité) et les variables dépendantes dont la couverture vaccinale, le refus vaccinal, l'hésitation vaccinale, l'intention vaccinale et les raisons de refus vaccinal contre la COVID-19. Les données quantitatives avaient été présentées sous forme de paramètres de position et de dispersion, tandis que les données qualitatives ont été présentées sous forme numérique absolue et relative. L'analyse des données avait commencé par le codage des questionnaires puis la description du profil sociodémographique des participants. La saisie et l'analyse des données avait été faites à l'aide du logiciel SPSS 26. Le logiciel Microsoft Excel avait été utilisé pour la confection des tableaux et graphiques. Les données manquantes ou incomplètes ont été considérées dans les analyses statistiques. Les mesures d'association entre les variables d'intérêt ont été effectuées à l'aide des tests de Chi-deux et de Fisher, au seuil d'erreur α=5% et au degré de significativité p < 5% et l'intervalle de confiance de 95%.

**Consentement éclairé:** le consentement éclairé des participants était le préalable pour être retenue pour l'étude. L'anonymat des participants était respecté. En effet les questionnaires étaient anonymes.

## Résultats

### Participants de l'étude

Nous avons rencontré 3532 personnes dont 225 non éligibles (175 non-résidents et 50 âgés de moins de 21 ans). Des 3307 éligibles, 1752 ont refusé de participer à cause du manque temps ou des raisons évoquées d'inexistence de la COVID-19 et de la théorie du complot. Seulement 1555 personnes ont été incluses dans l'étude, d'où un taux de recrutement égal à 44,02%.

### Couverture vaccinale contre la COVID-19

Concernant la couverture vaccinale contre la COVID-19, 168 sur 1555 interviewés soit 11% avaient reçu au moins une dose de vaccin contre la COVID-19.

### Refus vaccinal, hésitation vaccinale et intention vaccinale

Le Tableau 1 montre que 711 (45,7%) des participants avaient refusé le vaccin contre COVID-19. Par ailleurs, 640 (41,1%) des participants hésitaient à se faire vacciner contre COVID-19 et seulement 204 (13,2%) avaient l'intention de se faire vacciner contre la COVID-19.

**Tableau 1:** ampleur du refus vaccinal contre la COVID-19

Variables	Effectif N=1555	Proportion (%)
**Refus vaccinal**	711	45,7
Hésitation vaccinale	640	41,1
Intention vaccinale	204	13,2%
**total**	1555	100

### Raisons de refus vaccinal contre la COVID-19

Le Tableau 2 montre que les raisons principales de refus de vaccination contre COVID-19 sur les 907 personnes qui ont répondu étaient la crainte des effets indésirables 406 (44,8%), le manque d'information sur les vaccins 331 (36,5%) et le manque de confiance 302 (33,3%).

**Tableau 2:** raisons de refus et hésitation vaccinale

Raisons de refus vaccinal	Effectif (n=907)	Proportion (%)
Crainte des effets indésirables	406	44,8
Manque d’information sur les vaccins	331	36,5
Manque de confiance (Labo/autorités)	302	33,3
Ne pas vouloir être cobaye	156	17,2

n : effectif

### Facteurs associés au refus vaccinal

L'étude avait montré que 73% et 74% respectivement des femmes et hommes refusaient le vaccin anti-COVID-19 sans différence significative (p-value : 0,606). Il y avait une association entre la religion et le refus vaccinal contre la vaccination COVID-19 (p=0,026) et les musulmans sont ceux qui refusent le plus la vaccination 89 sur 105 soit 84,8%, suivi des protestants 315 sur 633 soit 74,4%. Il n'y avait pas d'association entre les groupes d'âges et le refus vaccinal contre COVID-19 (p=0,351). Cependant, on note que les personnes âgées de 65 ans et plus refusent le plus (79,9%). Quant à la profession, il n'y avait pas d'association entre les travailleurs et les sans-emplois (p=0,343) et le refus vaccinal même si on avait noté une plus grande proportion de 88,6% chez les travailleurs. Par ailleurs, l'étude avait montré qu'il existait une association entre le niveau d'étude te le refus vaccinal (p=0,002). Parmi les personnes ayant un niveau d'étude secondaire 374 sur 401 soit 93,3% refusaient la vaccination contre la COVID-19 suivies par celles du niveau universitaire 954 sur 1086 soit 88,7% (Tableau 3).

**Tableau 3:** associations entre le sexe, religion, groupe d'âges, l'emploi, le niveau d'étude et le refus vaccinal

variables			Effectif N=1555	Proportion (%)	p value
**Sexe**	Féminin	Non	624	73	0,606*
		Oui	23	27	
		Total	855	55,0	
	Masculin	Non	519	74 ,1	
		Oui	181	25,9	
		Total	700	45,0	
**Religion**	Protestant	Non	315	74,4	0,026*
		Oui	318	25,6	
	Catholique	Non	569	72,6	
		Oui	215	27,4	
	Musulmane	Non	89	84,8	
		Oui	16	15,2	
	pas de religion	Non	107	72,3	
		Oui	41	27,7	
	Autres	Non	63	74 ,1	
		Oui	22	24,9	
**Tranches d’âge**	21-34	Non	780	72,3	0,351*
		Oui	299	27,3	
	35-44	Non	149	74,5	
		Oui	51	25,5	
	45-64	Non	166	76,9	
		Oui	50	23,1%	
	65+	Non	47	79,7%	
		Oui	12	20,3	
**Profession**	**Travailleur**	Non	983	88,6	0,343*
		Oui	126	11,4	
	**Sans emploi**	Non	404	87,4	
		Oui	42	12,6	
**Niveau d’étude**	**Aucun**	Non	7	63,6	0,002*
		Oui	4	36,4	
	**Primaire**	Non	52	82,5	
		Oui	11	17,5	
	**Secondaire**	Non	374	93,3	
		Oui	31	7,7	
	**Universitaire**	Non	954	88,7	
		Oui	122	11,3	

* p-value significative

## Discussion

Les objectifs de l'étude ont été atteints. En effet, la couverture vaccinale était de 11%. Par ailleurs, l'ampleur du refus vaccinal, d'hésitation vaccinale et de l'intention vaccinale des participants dans la ville de Douala au Cameroun étaient respectivement de 711 (45,7%) ; 640 (41,1%), et 204 (13,2%). Ce qui avait confirmé l'es hypothèse de notre étude. Les principales raisons de refus vaccinal étaient la crainte vaccinale et le manque d'informations sur les vaccins contre la COVID-19. Les facteurs associés au refus vaccinal étaient la religion et le niveau d'étude.

### Couverture vaccinale contre la COVID-19

Un an après le début de la campagne de vaccination, peu de participants étaient vaccinés. Cette tendance est comparable aux personnes vaccinées dans toutes les autres villes du Cameroun à la même période [28,29]. La même tendance se retrouve la majorité des pays africains selon l'OMS et en Roumanie [26,27] ce qui est différent des pays comme l'Arabie Saoudite et le Paraguay où les couvertures vaccinales étaient respectivement de 86% et 93,1% [9,20].

### Refus vaccinal, hésitation vaccinale et intention vaccinale

Notre étude avait montré que 45,7% des participants avaient refusé le vaccin contre la COVID-19. Le refus vaccinal est également important dans certains pays comme le Chili et la Turquie où respectivement 28,6% et 33% des participants avaient refusé la vaccination contre la COVID-19 [18,19]. Notre étude avait montré que 41,1% des participants hésitaient à se faire vacciner contre la COVID-19. Ce résultat est différent et inférieur à ceux retrouvés par Menezes *et al*. Au Bénin, Libéria, Sénégal et Togo où 60% de la population hésitaient à se faire vacciner. Cependant Menezes *et al*. avaient également trouvé que moins de 20% de la population hésitaient dans certains pays comme le Nigeria, Kenya, Éthiopie, Niger, Ghana, Tunisie, Ouganda, Mozambique, Soudan, Burkina Faso, Afrique du Sud et Côte d’Ivoire [15]. En Bosnie Herzégovine, 74,3% de la population rejetaient ou hésitaient de se faire vacciner avec le vaccin contre la COVID-19 ce résultat suit la même tendance de notre étude [16]. Notre étude avait montré que 13,6% des participants avaient l'intention de se faire vacciner. Ce résultat est supérieur à celui de Salam *et al*. [25] en 2021 au Sénégal (23,7%) et inférieur à celui de Agbé *et al*. [26] en 2022 en Côte-d'Ivoire (52%) et de Bayle [24] en 2021 en France (66%).

### Raisons de refus vaccinal contre la COVID-19

Les principales raisons du refus vaccinal dans notre étude étaient la crainte des effets secondaires (44,8%), le manque d'information sur la vaccination (36,5%) et le manque de confiance aux laboratoires et autorités (33,3%). Certains auteurs ont trouvé les mêmes raisons principalement la crainte des effets indésirables et le manque de confiance aux laboratoires qui fabriquent les vaccins contre la COVID-19 dans certains pays de l'Afrique sub-saharienne comme le Burkina Faso, le Cameroun, l'Ouganda, la Sierra-Leone, le Rwanda, le Mozambique, au Chili, en Afrique du Sud, l'Allemagne, la Bosnie Herzégovine et la Turquie [15-19]. Au Mali et au Ghana Menezes *et al*. avaient montré que certaines personnes avaient une faible confiance au gouvernement et croyaient à la théorie complotiste [15].

### Facteurs associés au refus vaccinal

L'étude avait montré que la religion était associée au refus vaccinal ce qui est différent des travaux de de Bayle en France [24]. Dans notre étude, l'âge et le sexe n'étaient des facteurs associés au refus vaccinal contrairement au Sénégal [25]. Concernant l'intention vaccinale pour la vaccination contre la COVID-19, dans notre étude, les femmes avaient moins l'intention de se vacciner (11,6%) que les hommes (13%), avec une différence statistiquement non significative (p-value : 0,518). Nous avons trouvé une relation entre le niveau d'étude et le refus vaccinal, plus le niveau d'éducation était élevé plus le refus vaccinal était important. Cette tendance était la même en France [24].

### Limites

Du fait que c'était une étude transversale et une enquête CAP, on pourrait avoir des biais d'information. La taille des participants était appropriée donc nos résultats sont généralisables.

## Conclusion

L'étude avait montré que la couverture vaccinale contre COVID-19 était faible. La majorité des participants avaient refusé le vaccin contre COVID 19 et les principales raisons de refus vaccinal étaient la crainte et le manque d'informations sur les vaccins contre la COVID 19. Les facteurs associés au refus vaccinal étaient la religion et le niveau d'étude.

### Etat des connaissances sur le sujet

- *Le COVID-19 est une nouvelle maladie et la vaccination est un des moyens de prévention;*
- *Au Cameroun, l'expérience de la vaccination des adultes est rare en dehors des femmes enceintes qui reçoivent le vaccin contre le tétanos, voyageurs et les populations affectées lors des campagnes de vaccination en réponse aux épidémies causées par les maladies évitables par la vaccination;*
- *La couverture vaccinale COVID-19 est faible en Afrique*.

### Contribution de notre étude à la connaissance

- *L'ampleur du refus vaccinal, hésitation vaccinale et intention vaccinale des participants dans la ville de Douala au Cameroun étaient respectivement 711 (45,7%) ; 640 (41,1%), et 204 (13,2%)*.
- *Les principales raisons de refus vaccinal étaient la crainte vaccinale et le manque d'informations sur les vaccins contre la COVID-19;*
- *Les facteurs associés au refus vaccinal étaient la religion et le niveau d'étude*.

## Remerciements

Notre profonde reconnaissance à la population de la ville de Douala et aux autorités administratives de la ville de Douala et le corps enseignant de la Faculté de Médecine et des Sciences Pharmaceutiques de Douala.
